# Endocervical adenocarcinoma implantation in episiotomy scar: a case report and  review of the literature

**DOI:** 10.1186/s13256-023-03786-4

**Published:** 2023-03-08

**Authors:** Fariba Yarandi, Mahdi Aghili, Sara Ramhormozian, Elham Shirali

**Affiliations:** 1 Department of Gynecology Oncology, Yas Hospital, School of Medicine, Tehran University of Medical Sciences, Tehran, Iran; 2 Radiation Oncology Research Center, Cancer Institute, Imam Khomeini Hospital, School of Medicine, Tehran University of Medical Sciences, Tehran, Iran; 3 Department of Obstetrics and Gynecology, Yas Hospital, School of Medicine, Tehran University of Medical Sciences, Tehran, Iran

**Keywords:** Cervical cancer, Episiotomy scar adenocarcinoma, Chemoradiation, Disease-free survival, Pregnancy

## Abstract

**Background:**

Cervical cancer is a rare malignancy in the 1st months of pregnancy. Implantation of this cancer in an episiotomy scar is a condition that is rarely reported.

**Case presentation:**

We reviewed the literature on this condition and reported a 38 year-old Persian patient who had been diagnosed with cervical cancer, clinically stage IB1, 5 months after a term vaginal delivery. She underwent transabdominal radical hysterectomy with ovarian preservation. Two months later she presented with a mass-like lesion in the episiotomy scar which was proved to be of cervical adenocarcinoma origin after biopsy. The patient was scheduled for chemotherapy with interstitial brachytherapy, an alternative to wide local resection, with successful long-term disease-free survival.

**Conclusion:**

Implantation of adenocarcinoma in an episiotomy scar is a rare occurrence in patients with a history of cervical cancer and previous vaginal delivery near the time of diagnosis which requires extensive local excision as a primary treatment when feasible. The proximity of the lesion to the anus can lead to major complications of extensive surgery. Alternative chemoradiation combined with interstitial brachytherapy can be successful in eliminating cancer recurrence without compromising the functional outcome.

## Background

Cervical cancer is the most common gynecologic malignancy during pregnancy accounting for 71.6%, followed by ovarian malignant tumors, accounting for 7.0% [1]. The incidence of cervical cancer itself is not very high being about 0.05% among all pregnant women [2]. The implantation of malignant cells from cervical cancer in an episiotomy scar is a rare event by itself. In 1987 Burgess *et al.* [3] reported the first cervical cancer implantation in an episiotomy scar [2]. As we know, there are only twenty cases in literature resembling what we want to present. Here we report a case of adenocarcinoma implantation in episiotomy scar in a patient with a previous history of endocervical adenocarcinoma, which had undergone transabdominal radical hysterectomy with ovarian preservation 7 months before implantation. Besides, we will take a brief look at the review of literature in this field.

## Case presentation

A 38 year-old (gravida 1, para 1) Persian patient was referred to our oncology clinic at Yas hospital in May 2018. Due to persistent spotting 5 months past her last vaginal delivery a complete workup was done for her showing “atypical glandular cell (not otherwise specified)” [AGC-NOS] in her pap smear, and the human papillomavirus (HPV) test was also positive for HPV16. The transvaginal sonography showed a 1.3 cm pedunculated endocervical polyp with extension to exocervix. In the sliding maneuver, the cervical motion was limited. Mild adhesion in the posterior cul-de-sac with bilateral uterosacral ligament thickening was noted. Dilation & curettage plus endocervical curettage [D&C + ECC] was done for the patient with pathology report suggesting well-differentiated invasive adenocarcinoma with negative lymhovascular space invasion (LVSI). She also had a history of an endocervical polyp found accidentally during her last vaginal delivery which hadn’t been evaluated pathologically. The past medical history was unremarkable. A history of breast cancer and GI malignancy was found in the 2nd-degree family of the patient. Pre-operative pelvic magnetic resonance imaging (MRI) showed junctional zone thickening and myometrial heterogeneity, possibly due to adenomyosis. All other organs were normal. No ascites or lymphadenopathy was found. The patient was scheduled for surgery with diagnosis of cervical cancer clinically staged IB1. She underwent radical hysterectomy type 3 plus bilateral salpingectomy with ovarian preservation, and pelvic lymphadenectomy (bilateral common iliac, obturator, and external iliac lymph nodes) was done. The final pathology report showed moderately-differentiated endocervical adenocarcinoma (usual type-grade 2). Tumor size was measured 1.7 cm maximally originating from antero-inferior part of the cervix. It was positive for LVSI, but there was no perineural invasion. All 28 nodes were free from tumor. The vaginal margin was free, and no parametrial and paracervical involvement was identified. All other organs were unremarkable.

Around 7 months after surgery, she noticed a mass-like lesion in her episiotomy scar, growing over time. First, it was considered an inflammatory reaction, but reevaluation was done due to its gradual increase in size and pain. Due to adjacency of the lesion to the anal sphincter, wide local excision was avoided and incisional biopsy was done. The pathology report was ‘metastatic cervical adenocarcinoma.’ All the pathologic assessments of this patient was done by an experienced gynecologic pathologist to reduce interobserver bias and was reviewed by a second gynecologic pathologist for reassurance. In complementary immunohistochemistry (IHC) study, it was positive for EMA & CK7, negative for CK20 & weakly positive for CDX-2. In pelvic MRI, an oval superficially-located enhancing mass measuring 2.5*1.8*1.5 cm with well-defined border in the right perineal region was revealed about the posterior vagina and adjacent to external anal sphincter with close contact to right puborectalis muscle, external anal sphincter muscles and distal vagina. In metastatic workup, the chest and abdominal computed tomography (CT) scan was normal. Colonoscopy did not show any lesion in the anal canal, and the endosonography reported an ill-defined hypoechoic area with the upper part of the external sphincter (adjacent to the vagina). Due to the patient’s strong desire for organ-sparing treatment and incompliance with wide local surgery as primary treatment, chemo-radiotherapy was planned as the management strategy.

**Fig. 1:**
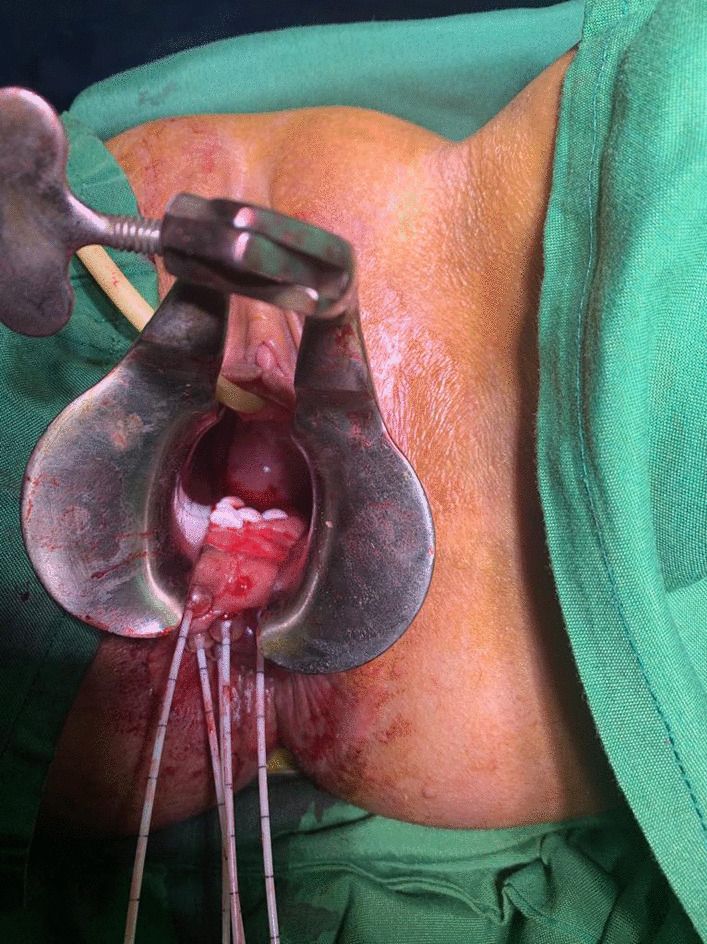
The lesion in gross view after insertion of interstitial plastic catheters in vaginal wall at the site of implantation

**Fig. 2:**
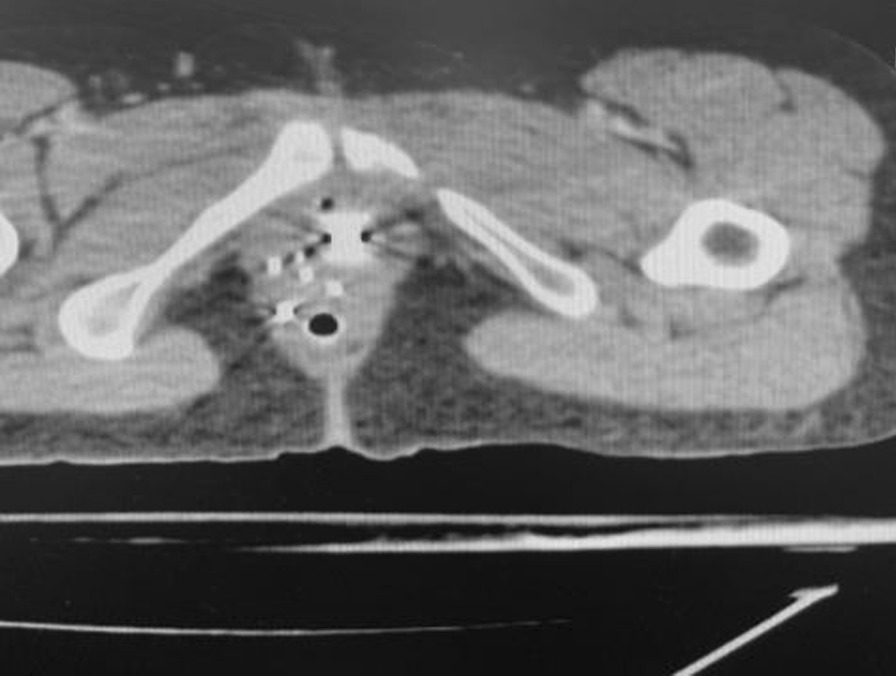
The axial view of the patient’s computed tomography scan after interstitial catheter insertion

**Fig. 3:**
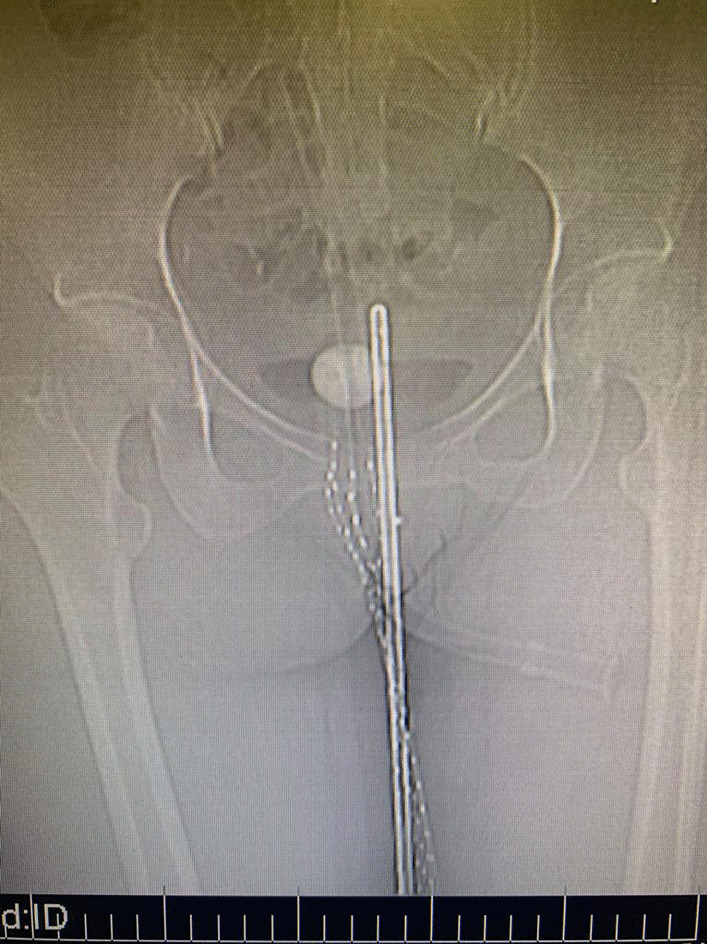
The pelvic anteroposterior plain radiography of the patient after interstitial catheter insertion

The patient underwent pelvic external radiation to cover the primary lesion, the cervical bed and the lymph nodes’ drainage system (including obturator, internal and external iliac, presacral and common iliac nodes up to the aortic bifurcation) for 50.4 Gy in 28 fractions concurrently with weekly chemotherapy with Cisplatin 40 mg/m^2^. One week later the patient underwent interstitial brachytherapy by insertion of five interstitial plastic catheters in vaginal wall at the site of implantation (Fig. 1). After CT scan planning (Figs. 2, 3), the images were transferred to the treatment planning device using the Flexiplan® software. The clinical target volume (CTV) was defined as the primary tumor at the time of implantation with 5–10 mm margin around it excluding the rectum and the bladder which were defined as organs at risk. The CTV was treated to a total dose of 21 Gy in 7 fractions with 2 fractions delivered per day and a 6-hours interval between fractions. Treatment was carried using BEBIGS® high dose rate (HDR) brachytherapy unit with cobalt-60 radioactive source.

Since the last treatment session, the patient has been under close follow up. She has been examined every 3 months in the clinic with cervical cytology, physical examination and MRI. In the last follow-up visit in December 2022, she is free of disease for more than 3 years without major treatment adverse effects like proctitis, cystitis or vaginal stenosis with good anal sphincter function.

## Discussion and conclusions

Implantation of cervical carcinoma in the episiotomy scar is a rare condition. The first case was reported by Burgess *et al.* [3] in 1987. She was a 33-year-old woman with implantation of previously diagnosed cervical squamous carcinoma in episiotomy scar 21 months after initial malignancy diagnosis. The last and 20th case was described by Carocha *et al.* [4] in 2015. The interval between initial diagnosis and the implantation was 3 months, with histopathology of Glassy Cell Carcinoma. Our patient is the 21st patient with implantation of cervical cancer in episiotomy scar. The patient presented by the authors is the oldest patient reported in the literature. The mean age of previous cases was 33.3, ranging from 21 to 37. The interval from the primary cervical cancer diagnosis and the implantation had a wide range, from 5 weeks to 5.5 years. Like most previous reports, our patient was diagnosed with cervical cancer stage IB1, according to the 2018 International Federation of Gynecology and Obstetrics (FIGO) staging system. Due to the rarity of cases, treatment is controversial, from chemoradiation to abdominoperineal resection. In previously reported patients, the metastatic lesion in the episiotomy scar was described as a nodular, ulcerative, or cystic lesion. Swelling, granulation, or abscess formation was also reported as the primary complaint of the patient. The size of the metastatic lesion ranged from 0.5 to 5 cm in the literature. Our patient had a mass-like lesion in her episiotomy scar, with a 2.5 cm diameter. The information about previous cases is summarized in Table 1.

**Table 1:** The list and characteristics of the same case reports in the literature

Author	Age	Time of initial cervical cancer diagnosis	Histological type	Initial stage of cervical neoplasia	The interval from the initial diagnosis to detection of recurrence	Size of recurrence	Treatment	Outcome
Khalil * et al.* [5]	32	8 week postpartum	Squamous carcinoma	IIIB	3 months	40	Chemotherapy and radiotherapy	Died of disease (4 months)
Cliby * et al.* [6]	37	Biopsy at delivery	Squamous carcinoma	IB	9 weeks	N/A	Chemotherapy	Died of disease (6 months)
Cliby * et al.* [6]	31	Cervical lesion at 36 weeks; biopsy at delivery	Squamous carcinoma	IB	2 years	N/A	Excision, radiotherapy, and chemotherapy	Disease-free (12 months)
Cliby * et al.* [6]	21	At delivery	Squamous carcinoma	IB	3 months	N/A	Radiotherapy	Died of disease (36 months)
Cliby * et al.* [6]	34	5 weeks postpartum	Squamous carcinoma	IB	1 month	N/A	Excision and radiotherapy	Died of disease (6 months)
Van den Broek *et al.* [7]	29	3 month s postpartum	Squamous carcinoma	IA	6 weeks	15	Chemotherapy and radiotherapy	Died of disease (12 months)
Sood * et al.* [8]	N/A	N/A	N/A	IIA	5 months	N/A	Radiotherapy	Died of disease (N/A)
Goldman *et al.* [9]	35	Lesion at 35 weeks; biopsy 1 week postpartum	Adenocarcinoma	IB	5.5 years	50	Excision and radiotherapy	Disease-free (54 months)
Amanie * et al.* [10]	30	1.5 years postpartum	Adenocarcinoma	IIIA	0	43*32	Chemotherapy and radiotherapy	Disease-free (12 months)
Heron * et al.* [11]	32	Biopsy at delivery	Adenocarcinoma	IB	4 years	20*20*10	Radiotherapy and excision	Disease-free (48 months)
Baloglu * et al.* [12]	36	8 months postpartum	Squamous carcinoma	IIIA	0	40	Chemotherapy and radiotherapy	Disease-free (12 months)
Neumann * et al.* [13]	35	At delivery	Adenosquamous	IB	5 weeks	50*60	Excision, chemotherapy, and radiotherapy	Died of disease (8 months)
Hafeez * et al.* [14]	34	At delivery	Squamous carcinoma	IB	5 months	N/A	Chemotherapy and radiotherapy	Disease-free (120 months)
Carocha * et al.* [4]	34	4 weeks postpartum	Cervival Glassy cell carcinoma	IB	3 months	50	Excision, radiotherapy, and chemotherapy	Died of disease (9 months)

Lower vaginal cervical metastasis is not common in the natural history of cervical malignancy [10]. The implantation may occur during vaginal delivery rather than vascular or lymphatic metastasis [15]. All of the cases reported in the literature have a history of episiotomy or traumatic lacerations. The pathogenetic mechanism suggested in the literature is similar to that of endometriosis implantation. The mechanism of episiotomy recurrence is local implantation of tumor cells rather than lymphatic or haematogenous spread. Factors that support the implantation hypothesis include similarity of pathology and immunohistochemistry findings between primary tumor and episiotomy scar metastasis, history of grade 3 vaginal tear, and the rarity of skip vaginal metastases in cervical cancer [10]. Han *et al.* [16] reported a case of primary clear cell carcinoma developing within a previous episiotomy scar in a patient with a history of endometriosis. They also stated that implantation or transport theory is the best explanation of endometriosis at episiotomy scar. Due to the possibility of malignant cell implantation, the cesarean section should be the preferred method of delivery rather than vaginal delivery [1, 17].

The most important strategy to prevent vaginal metastasis is early diagnosis and treatment of primary cervical malignancy. Some obstacles exist for early diagnosis of cervical cancer in the pregnancy: First, prenatal cervical screening is often ignored by pregnant women. Second, vaginal discharge and bleeding are common complaints during pregnancy, which may be confused with different obstetric complications and eventually lead to underdiagnosis of the cervical pathology. Third, detecting a cervical abnormality while doing colposcopy is difficult in pregnancy and needs technical expertise due to reasons such as: increased cervical mucus production which obscures visualization, cervical hyperemia, gland prominence, and eversion of the columnar epithelium [18]. Fourth, many patients have a wrong belief that performing a vaginal and cervical examination during pregnancy is dangerous [19]. A careful vaginal examination during pregnancy and at the time of labor could provide a diagnosis of gross cervical lesions. Because of the high prevalence of cervical cancer in developing countries, prenatal care should be considered the best chance for cervical cancer screening. Finding gross cervical lesions, changes the delivery mode. Any irregular vaginal bleeding, discharge, and pain or mass at the episiotomy site during the postpartum period should warn us of the possibility of cervical cancer. Also, Patients with the diagnosis of cervical malignancy during pregnancy or after vaginal delivery need a longer follow-up due to the possibility of metastasis in the episiotomy site [1, 12, 19].

The treatment strategies are different in the literature, including surgical management, systemic chemotherapy, and radiotherapy or combinations of them. Nevertheless, if possible, it seems that a combination of wide local excision with free margins followed by radiotherapy may lead to better outcomes [9, 13]. The technique and the extent of the surgery has not been defined precisely in the literature and is varied case by case. The treatment of choice for our patient was radical surgery but she refused surgical intervention. Chemoradiotherapy combined with interstitial brachytherapy was undertaken as an alternative for surgery. The use of definitive chemoradiation with or without brachytherapy is an optional treatment alternative to local excision with/without adjuvant radiotherapy with contradictory results. Some of these patients had vaginal tumor concomitant with cervical cancer and some recurrent in episiotomy site after primary treatment of cervical cancer. In our report we used interstitial plastic tube for HDR treatment in outpatient setting with the advantages of 3 dimensional treatment planning in recurrent tumor after primary treatment in episiotomy site in contrast to the other reports [5, 7, 10, 12] and with long term disease free survival like the report of Hafeez *et al.* [14] that was treated only by external radiotherapy (photon + electron) although she had 10 years disease free survival but at the cost of high complications of perineal cellulitis and obstruction uropathy that required continued management.

In conclusion, implantation of adenocarcinoma in an episiotomy scar is a rare occurrence in patients with a history of cervical cancer and previous vaginal delivery near the time of diagnosis which requires extensive local excision as a primary treatment when feasible. This emphasizes the importance of careful postoperative perineal inspection. The proximity of the lesion to the anus can lead to major complications of extensive surgery in many cases. Alternative chemoradiation combined with interstitial brachytherapy can be successful in eliminating cancer recurrence without compromising the functional outcome. After the latter treatment modality in our report, the patient did not experience any serious and long-term complications; however, due to small data and lack of randomized trials, decision making about the best treatment method should be individualized based on many factors such as the patient’s preference, life expectancy, stage of current disease, location of the implantation and the surgeons’ experience and making a safe conclusion is impossible.

## Data Availability

Data sharing does not apply to this article as no new data were created or analyzed in this study.

## Abbreviations

AGC-NOS: Atypical glandular cell-not otherwise specified
HPV: Human papilloma virus
D&C: Dilation and curettage
ECC: Endocervical curettage
LVSI: Lymphovascular space invasion
MRI: Magnetic resonance imaging
IHC: Immunohistochemistry
CT: Computed tomography
CTV: Clinical target volume
HDR: High dose rate
EQ: Equivalent dose
FIGO: International Federation of Gynecology and Obstetrics
